# Neurodegenerative Disease: From Molecular Basis to Therapy

**DOI:** 10.3390/ijms25020967

**Published:** 2024-01-12

**Authors:** Claudia Ricci

**Affiliations:** Department of Medical, Surgical and Neurological Sciences, University of Siena, 53100 Siena, Italy; claudia.ricci@unisi.it

Neurodegenerative diseases are a heterogeneous group of age-related disorders characterised by the progressive degeneration or death of neurons in the central or peripheral nervous system. The prevalence of these diseases is increasing—in part due to the ageing population—and the economic burden on healthcare systems is growing as a result. Although in some cases these diseases can be managed with treatments, current therapies are mostly symptomatic, do not address the underlying cause of the disease and have very little effect on disease progression. Recent advances in neurobiology and neurogenetics have provided valuable insights into the pathogenesis of neurodegenerative diseases. Genetic, environmental and lifestyle factors contribute to neurodegenerative diseases. Common underlying processes contribute to the degeneration of neurons, but the molecular mechanisms of neurodegenerative diseases are complex and diverse and may differ between conditions. As progress is made in understanding critical aspects of the underlying molecular pathophysiology, therapeutic strategies are evolving and new treatments are being evaluated.

Several papers in this Special Issue focus on Alzheimer’s disease (AD), the most common cause of dementia, characterised by memory loss, behavioural disturbances and impaired judgment [1]. The presence of senile plaques, characterised by the aggregation of amyloid beta (Aβ), and the formation of neuronal neurofibrillary tangles (NFTs) are well-known neuropathological hallmarks of AD [2]. Symptoms of AD generally begin with mild memory impairment and progress to various degrees of severe cognitive impairment, including memory loss and difficulty with complex activities of daily living [3,4]. These pre-dementia stages could play a key role in preventive interventions: the development of early and accessible diagnostic methods could help to prevent or delay the progression of cognitive deficits and the onset of AD dementia [5,6].

In this respect, Hunjong Na and colleagues [7] developed the QPLEX™ kit for the early clinical diagnosis of Alzheimer’s disease. This kit simultaneously detects amyloid-β1-40, galectin-3 binding protein, angiotensin-converting enzyme and periostin in a few microlitres of peripheral blood and uses an optimised algorithm to screen for AD by correlating with cerebral amyloid deposition. The authors evaluated cognitively normal subjects and patients with subjective cognitive decline, mild cognitive impairment and AD, and showed that the QPLEX™ algorithm values could be used to distinguish the clinical continuum of AD or cognitive function. The QPLEX™ kit could be a valuable tool for health screening and early clinical diagnosis of Alzheimer’s disease, as blood-based diagnosis is more accessible, convenient and cost- and time-effective than diagnosis based on cerebral spinal fluid or positron emission tomography.

Another important aspect of dementia research is the development of new therapeutic approaches aimed at curing the disease or alleviating its symptoms. In their research, Nicole E. Eassa and colleagues [8] focused on one of the most common comorbidities associated with Alzheimer’s disease, comorbid psychosis, which affects half of all AD patients [9]. Since it is not possible to treat elderly patients with antipsychotics due to an increased risk of premature death, there is a clear need for novel therapeutic options for AD patients with comorbid psychosis. The authors used a viral-mediated approach to express mutated human genes known to contribute to Alzheimer’s pathology in a rat model. The authors observed a significant increase in dopamine neuron population activity and behavioural deficits in rodent models of psychosis-like symptomatology. Furthermore, systemic administration of an α5-GABAA receptor-selective positive allosteric modulator was able to reverse the aberrant function of the dopaminergic system in AD-AAV rats. This study provides interesting insights for the development of drugs targeting α5-GABAA receptors for patients with Alzheimer’s disease and comorbid psychosis.

Hoau-Yan Wang and colleagues [10] investigated the molecular mechanisms of simufilam, a novel oral drug candidate in phase 3 clinical trials for Alzheimer’s dementia. Simufilam is a small molecule that binds an altered form of filamin A (FLNA) found in AD. This binding disrupts FLNA’s aberrant binding to the α7 nicotinic acetylcholine receptor (α7nAChR), thereby blocking soluble amyloid beta1-42 (Aβ42) signalling via α7nAChR, which hyperphosphorylates tau. The authors showed that simufilam reduces Aβ42 binding to α7nAChR. They also showed that FLNA binds to several inflammatory receptors in addition to Toll-like receptor 4 (TLR4) in postmortem human AD brains and in AD transgenic mice. These aberrant FLNA connections were disrupted by simufilam. Simufilam also reduced inflammatory cytokine release from Aβ42-stimulated human astrocytes. Taken together, these data suggest that simufilam may promote brain health by disrupting abnormal FLNA receptor interactions that are critical for AD pathogenic pathways.

Two reviews summarise the state of the art in understanding the molecular mechanisms responsible for the development of AD and consequently identifying novel drug targets. In their manuscript, Botond Penke and colleagues [11] accurately describe the pathogenesis of AD, its genetic background and the physiological and pathophysiological roles of the Aβ and Tau proteins. They also discuss the various hypotheses that have been proposed to explain AD neurodegeneration. In the second part of the paper, they summarise conventional and novel targets for preventing and/or slowing the progression of AD. The authors conclude that it is not possible to treat all stages of AD with a single drug. A drug combination strategy with multiple molecular targets (amyloid aggregation, clearance, heat shock proteins, autophagy induction, inflammasomes, etc.) should be considered.

The paper by Kseniia Orobets and Andrey Karamyshev [12] reviews the current knowledge on amyloid precursors and Alzheimer’s disease. Amyloid precursor protein (APP) is a membrane protein that is thought to play a major role in the pathology of AD. APP is known to follow a non-amyloidogenic pathway under physiological conditions; however, it can progress to an amyloidogenic scenario, leading to the formation of extracellular deleterious Aβ plaques. The authors summarise the biogenesis, processing and mechanisms of action of APP. They conclude that despite decades of research on Alzheimer’s disease and APP, not all steps of APP biogenesis have been elucidated and that many questions about APP biogenesis, especially the early steps, interacting partners, the role of APP in microtubules and the potential therapeutic targets, need to be addressed in future studies.

Finally, in their Perspectives paper, Vladimir Volloch and Sophia Rits-Volloch [13] have comprehensively reviewed the molecular basis and potential effective therapies of the Amyloid Cascade Hypothesis 2.0 (ACH2.0) for Alzheimer’s disease and age-related cognitive decline (AACD). ACH2.0 is a recently proposed theory of Alzheimer’s disease. Its name refers to its predecessor, ACH, although the similarity between the two theories is limited to the recognition of the centrality of amyloid-beta (Aβ) in the disease. In ACH, the disease is caused by secreted extracellular Aβ, while in ACH2.0, it is triggered by Aβ protein precursor (AβPP)-derived intraneuronal Aβ (iAβ) and driven by iAβ generated independently of AβPP. ACH2.0 considers AD as a two-stage disease. In the first, asymptomatic stage, there is a decades-long accumulation of AβPP-derived iAβ via internalisation of secreted Aβ and intracellular retention of a fraction of Aβ produced by AβPP proteolysis. When AβPP-derived iAβ reaches critical levels, it activates a self-sustaining AβPP-independent production of iAβ that drives the second, devastating stage of AD, involving tau pathology and culminating in neuronal loss. The authors analyse the dynamics of iAβ accumulation in health and disease and identify it as a major driver of both AD and age-related cognitive decline. They discuss the mechanisms potentially involved in the AβPP-independent generation of iAβ and provide mechanistic interpretations for all major aspects of AD and AACD. They conclude that drugs that affect the accumulation of AβPP-derived iAβ may only have a protective effect on AD, whereas targeted degradation of iAβ is the best therapeutic strategy for both prevention and effective treatment of AD and AACD.

Another substantial part of this Special Issue is dedicated to Parkinson’s disease (PD). PD is the second most fatal neurodegenerative disorder, identified by neuronal degeneration in the substantia nigra pars and intracellular deposition of Lewy bodies [14]. There is still no clear understanding of the aetiology of Parkinson’s disease, and existing treatments only provide effective control of symptoms, without stopping the disease from progressing. Therefore, there is a great need for therapeutic strategies to reduce neuronal death.

One of the possible strategies is to reduce the level of accumulated alpha-synuclein (SNCA) in neurons. Autophagy is the main cellular process for removing toxic protein aggregates responsible for neurodegenerative diseases. It is a complex process involving dozens of proteins [15]. Ibrar Siddique and colleagues [16] have identified a novel regulator (ARL6IP5) of neuronal autophagy whose levels decrease in the brain with age and in Parkinson’s disease. Overexpression of ARL6IP5 reduces α-synuclein aggregation and improves cell survival in a mouse model of PD. Interestingly, they showed that ARL6IP5 is an autophagy inducer that enhances autophagosome initiation and elongation. In addition, they showed for the first time that α-synuclein downregulates ARL6IP5, thereby inhibiting autophagy-dependent clearance of toxic aggregates and exacerbating neurodegeneration. Taken together, these results suggest the potential of ARL6IP5 as a target for diseases in which autophagy is deregulated.

In their study, Prachayaporn Prasertsuksri and colleagues [17] have shown that andrographolide (andro) has significant neuroprotective effects against 1-methyl-4-phenylpyridinium (MPP+), a neurotoxin that can cause loss of dopaminergic neurons, in SH-SY5Y cells. Andro reduces cell death by increasing mitophagy and autophagic clearance of alpha-synuclein and by increasing the antioxidant capacity. These results provide evidence that andro could be considered as a potential supplement for the prevention of Parkinson’s disease.

SH-SY5Y cells were also used as a model of dopaminergic neurons in the study by Wei Zheng and colleagues [18]. U251 cells were used as a model for astrocytes. The study was designed to investigate the role of leukocyte common antigen-related protein tyrosine phosphatase (LAR) in Parkinson’s disease. LAR knockout showed a protective effect in astrocytic cells, reducing cell death and restoring an appropriate cell morphology. The basis of this effect was an enhanced neuroprotective capacity due to the activation of IGF-1R and Akt and the consequent reduction in the Bax/Bcl-2 ratio and suppression of apoptosis. Akt also drove the upregulation of NRF2 and HO-1, resulting in the suppression of ROS production, the preservation of mitochondrial function and increased GDNF production. Higher levels of astrocytic viability and GDNF production also contributed to the increased viability of co-cultured SH-SY5Y neuronal cells in a PD model system. Taken together, these findings suggest that inhibition of LAR may modulate astrocyte viability and function and may provide a novel therapeutic strategy for PD.

Finally, Thomas Stojsavljevic and colleagues [19] focused on another type of therapeutic approach to PD, deep brain stimulation (DBS), which consists of surgically implanting an electrode in the subthalamic nucleus (STN) to send electrical impulses to the targeted regions of the brain. Since the conventional high-frequency (HF) stimulation currently used as standard has several drawbacks, the authors carried out a computational study to overcome the limitations of HF stimulation. The authors stimulated the STN in an adaptive manner, using the interspike time of the neurons to control the stimulation. This protocol eliminated bursts in the synchronised bursting neuronal activity of the STN, which can cause thalamocortical (TC) neurons to fail to respond properly to excitatory cortical inputs. It also significantly reduced TC relay errors, showing promise for future Parkinson’s disease therapeutics.

Among neurodegenerative diseases, amyotrophic lateral sclerosis (ALS) is a rapidly progressive and fatal disorder characterised by progressive degeneration of upper and lower motor neurons in the cerebral cortex, brainstem and spinal cord. The pathophysiological process underlying ALS neurodegeneration is multifactorial and still not fully understood, although dysfunctions in several cellular and molecular processes have been reported, including impaired protein homeostasis, mitochondrial alterations, aberrant RNA metabolism, neuroinflammation, excitotoxicity and oxidative stress [20]. Several studies have implicated aberrant regulation of PKC-mediated signalling pathways in ALS through alterations in either the expression or activity state of several members of the PKC superfamily [21,22]. Valentina La Cognata and colleagues [23] analysed the distribution and cellular localisation of the ε-isozyme of protein kinase C (PKCε), a novel isoform of PKC that represents an attractive target for the treatment of several conditions, including neurodegenerative diseases. In human postmortem motor cortex samples, they found a significant decrease in both PKCε mRNA and protein immunoreactivity in a subset of sporadic ALS patients (ALS-GLIA, defined by an increased expression of genes that mark astrocytes and oligodendrocytes [24]). In addition, NSC-34 cells carrying the human G93A SOD1 mutant exhibited a significant reduction in the phosphoPKCε/panPKCε ratio compared to WT cells. Furthermore, a short pulse activation of PKCε by its agonist Bry-ostatin-1 produced a long-term neuroprotective effect in degenerating G93A SOD1 cells. Taken together, these data support the involvement of PKCε in the ALS pathophysiology and suggest its pharmacological modulation as a potential neuroprotective strategy, at least in a subset of sporadic ALS patients.

Cássia Arruda de Souza Pereira and colleagues [25] focused on Huntington’s disease (HD), a progressive neurodegenerative disorder characterised by motor changes, progressive cognitive loss and psychiatric disorders. HD is caused by a mutation in the gene encoding huntingtin (Htt), which results in an expansion of the CAG trinucleotide, leading to abnormal long repeats of polyglutamine (poly-Q) in the N-terminal region of huntingtin which form abnormal conformations and aggregates. HD is also associated with deregulation of calcium (Ca2+) signalling and homeostasis [26,27]. Intracellular Ca2+ is stored in lysosomes, organelles involved in endo-cytic and lysosomal degradation processes, including autophagy [28]. Nicotinic acid adenine dinucleotide phosphate (NAADP) is an intracellular second messenger that promotes Ca2+ release from the endo-lysosomal system via activation of two-pore channels (TPCs) [29]. The authors have shown that in murine astrocytes overexpressing mHtt-Q74, mHtt-Q74 colocalises with the TPC2 receptor in lysosomes, thereby interfering with the physiological function of this channel and inducing Ca2+ re-release from these organelles. Increased Ca2+ levels from the lysosome in turn promote mHtt-Q74 aggregation. Furthermore, autophagy is inhibited in astrocytes overexpressing mHtt-Q74. These results support the hypothesis that lysosomal homeostasis is important in inhibiting mHtt aggregation and highlight the role of autophagy in neuroprotection.

In their concept paper, Dieu Thao Nguyen and colleagues [30] address the issue of obstetric neuropathy in diabetic patients. They propose a “two-hit” model to explain the effects of diabetes on mothers who are already in a putative subclinical state of damage and then experience neuronal damage during childbirth. Pregnant women with diabetes have a damaged nervous system, although the condition may be subclinical: this is the “first hit”. The process of childbirth can cause damage to the mother’s nervous system during delivery, which is the “second hit”. The authors describe the different pathological processes responsible for worsening neuropathy in diabetes mellitus and highlight the risk of obstetric neuropathy.

Two reviews examine the role of appropriate micronutrient supplementation on the nervous system. Zhengyang Quan and colleagues [31] describe the effect of a balanced intake of macro-, micro- and trace elements to improve and/or reduce the risk of depression. They discuss the effects of glucose, fatty acids, amino acids and mineral elements such as lithium, zinc, magnesium, copper, iron and selenium. These elements exacerbate or alleviate depression by regulating a range of physiological processes, including neural signal transmission, inflammation, oxidative stress, neurogenesis and synaptic plasticity. The paper also looks at the balance of these nutrients in the body, describing the effects of both nutritional deficiencies and episodes of depression.

In their review, Bianca Caroline da Cunha Germano and colleagues [32] summarise the scientific evidence on the effects of vitamin E supplementation on neuroprotection and neurodegeneration in experimental models. Vitamin E supplementation significantly improves memory, cognition, learning, motor function and brain markers associated with neuroregeneration and neuroprotection. It also reduces beta-amyloid (Aβ) deposition and toxicity in experimental models of Alzheimer’s disease. Furthermore, it reduces tau protein hyperphosphorylation and increases the levels of superoxide dismutase and brain-derived neurotrophic factor (BDNF) in rodents. For these reasons, the use of vitamin E could prevent and/or delay the progression of degenerative lesions in the central nervous system.

Finally, three papers address the general mechanisms involved in neurodegeneration. In their original article, Paul A. Hyslop and colleagues [33] discussed the origin of elevated S-glutathionylated GAPDH in chronic neurodegenerative diseases. Using biochemical and in silico molecular dynamics simulations, their study elucidates which factors may contribute to the persistence of S-glutathionylated GAPDH in different pathophysiological conditions. The results of the research provide a molecular rationale for how oxidative stress elevates S-glutathionylated GAPDH in neurodegenerative diseases and suggest novel targets for therapeutic intervention.

Alexander Pilski and Steven M. Graves [34] focused on the effects of repeated methamphetamine (meth) administration on substantia nigra pars compacta (SN) and locus coeruleus (LC) neurons in a mouse model. They observed that repeated meth exposure produced SN and LC axonal deficits prior to somatic loss in males, consistent with a dying-back pattern of degeneration, whereas female mice were resistant to chronic meth-induced degeneration. Interestingly, the pattern of degeneration observed in male mice and the sex difference paralleled in Parkinson’s disease [35] and patients with a history of meth abuse have been reported to lead to an increased risk of developing Parkinson’s disease [36]. In addition, exposure to meth also increases the risk of Alzheimer’s disease, as LC degeneration has been linked to pathogeneses associated with this disease [37]. Taken together, these findings suggest that the adverse effects of meth abuse may extend beyond neurotoxicity and represent a potential risk for the development of neurodegenerative diseases. This risk may be restricted to males, as female mice were resistant to meth-induced neurodegeneration.

Finally, the review by Jun-Hao Wen and colleagues [38] recapitulates the main aspects of a topic strictly related to neurodegeneration: cellular protein aggregation. They reviewed the composition and causes of protein aggregation in mammalian cells and summarised the damage caused by protein aggregates, describing how these aggregates affect various cellular functions. They have also highlighted some of the clearance mechanisms involved in removing the aggregates and discussed potential therapeutic strategies targeting protein aggregates in the treatment of ageing and age-related neurodegenerative diseases. Considering that the achievement of this goal requires a more comprehensive understanding of the organisation and relationship between protein homeostasis and protein aggregation, the authors conclude that further studies will be fundamental to follow this path.

In conclusion, this Special Issue highlights the continuing efforts of the scientific community to unravel the pathophysiological mechanisms responsible for neurodegeneration and to identify potential treatments for neurodegenerative diseases. The aim of this research is to understand the molecular basis of disease onset and progression, to obtain an earlier diagnosis, to identify novel therapeutic targets and ultimately to develop more effective therapies to counteract the progression of these still fatal diseases.
